# The role of coinhibitory receptor-expressing non-T cells in inflammation and immunity: unsung heroes or peripheral players?

**DOI:** 10.1038/s12276-025-01562-6

**Published:** 2025-11-03

**Authors:** Chaimae Khaled, Mijin Kim, Booki Min

**Affiliations:** 1 Department of Microbiology and Immunology, Northwestern University Feinberg School of Medicine, Chicago, IL USA; 2 Center for Human Immunobiology, Northwestern University Feinberg School of Medicine, Chicago, IL USA

**Keywords:** Cellular immunity, Monocytes and macrophages

## Abstract

Immune responses are finely regulated by multiple mechanisms, among which immune regulatory coreceptor family molecules play a central role in both enhancing and suppressing immune responses. Traditionally, T cells have been considered the primary cell type expressing these receptors, through which their responses are modulated. This understanding led to the emergence of the field of ‘immune checkpoint blockade’, which aims to rejuvenate T cells that have become exhausted in the context of chronic infections or the tumor environments. The molecules targeted by such approaches include PD1, CTLA4, Lag3, Tim3 and TIGIT, coinhibitory receptors predominantly expressed on conventional T cells exhibiting functionally impaired, exhausted phenotypes. Interestingly, an expanding array of non-T cell types also express these receptors, although their specific roles remain largely elusive. Here we explore the immune regulatory functions of these coreceptors as expressed on non-conventional T cells, such as myeloid cells and B cells, highlighting their potential contributions to immune regulation.

## non-T cell immune checkpoints shape immune responses

The immune system is a complex network of cells and molecules that protect the host from infection and disease. Dysregulation of these processes can result in pathological conditions, including chronic infections or cancer. Coinhibitory receptors such as PD1, CTLA4, Lag3, Tim3 and TIGIT are well established as key regulators of T cell-mediated immune responses. Emerging evidence indicates that these receptors are also expressed on non-T cell populations, including myeloid lineage cells and B cells, although their functional contributions in this context remain poorly understood. This review summarizes current knowledge on the expression and function of coinhibitory receptors in non-T cells and discusses how their therapeutic targeting beyond T cells may offer novel opportunities for restoring immune function in cancer and other diseases.

This summary was initially drafted using artificial intelligence, then revised and fact-checked by the author.

## Introduction

Immune responses are regulated by highly complex processes at multiple levels^1,2^. In case of T cells, T cell-receptor recognition of specific peptides presented by self-MHC molecules on antigen presenting cells ensures the antigen-specific T cell expansion and effector function^3^. The responses are further shaped by secondary and tertiary signals, known as costimulatory and cytokine stimulation, which enable activated T cells to clonally expand and differentiate into effector T cell subsets with distinct functions, particularly in the case of helper CD4^+^ T cells. Secondary costimulatory molecules are instrumental in supporting IL2 production and proliferation, with CD28 being the founding member, establishing key principles of clonal expansion, differentiation, as well as nonresponsiveness such as anergy, all of which are critical for effective immune responses and peripheral tolerance^4^. Tertiary cytokine signals typically derived from T cell-extrinsic sources induce master transcriptional programs capable of orchestrating T cell differentiation processes^5^. T cells activated in the presence of antigen presenting cell-derived IL12 become IFNγ-producing Th1 type effector cells specialized in activating macrophages and eliminating intracellular pathogens^6,7^. T cells activated with IL4 possibly provided by basophils and dendritic cells instead become IL4/IL13-producing Th2 type effector T cells that mount anti-helminth immunity^8,9^. The presence of IL6 plus TGFβ supports effector T cells exhibiting IL17-producing Th17 type lineage phenotypes essential for anti-extracellular pathogens and autoimmune inflammation^10,11^. Activated CD8^+^ T cells predominantly become cytotoxic effector cells capable of eliminating virus infected or transformed cells.

Effector immune responses are tightly regulated by counterbalancing mechanisms, and coinhibitory receptors play a central role in preventing excessive activation. A growing number of inhibitory coreceptor families have been identified, and much of the research so far has focused on their ability to suppress T cell activation^12^. In chronic infections and tumor settings, dysfunctional T cells that highly express these coinhibitory receptors are found^13,14^. These so-called exhausted T cells display impaired effector functions, contributing to pathogen persistence and tumor immune evasion. To counteract this, immunotherapies targeting coinhibitory receptors have been developed, aiming to reverse T cell exhaustion, restore effector functions and ultimately promote pathogen clearance and tumor eradication.

Although conventional effector T cells are the most well-characterized cell type expressing coinhibitory receptors, a variety of other immune cell types also express these receptors, though their functions remain relatively poorly understood. This review will provide an overview of the biology of key coinhibitory receptors—cytotoxic T-lymphocyte antigen 4 (CTLA4), lymphocyte activation gene 3 (Lag3), programmed cell death protein 1 (PD1), T cell immunoglobulin and mucin domain 3 (Tim3) and T cell immunoreceptor with Ig and ITIM domains (TIGIT)—and examine their roles in non-conventional cell types.

## PD1

PD1 was discovered in IL3-deprived Ly9D (a murine hematopoietic progenitor cell line) and activated 2B4-11 (a T cell hybridoma cell line) cells that undergo programmed cell death^15^. PD1, along with CTLA4, is a prototype coinhibitory receptor that suppresses T cell activation and their effector function^16^. Upon engagement with its ligands, PDL1 or PDL2, PD1 undergoes phosphorylation at two conserved tyrosine residues located within the cytoplasmic domain: the immunoreceptor tyrosine-based inhibitory motif (ITIM) and the immunoreceptor tyrosine-based switch motif (ITSM)^17^. The phosphorylated motifs recruit the phosphatases SHP1 and SHP2, which then dephosphorylate key signaling intermediates downstream of the TCR and CD28 costimulatory pathway^18^. By dephosphorylating kinases such as ZAP70, PI3K and RAS, PD1 inhibits multiple downstream signaling pathways, reducing the activation of key transcription factors, including NFAT, AP1 and NFκB, thereby suppressing immune responses^19^.

Besides interfering with activation signaling pathways, PD1 signaling also shifts T cell metabolism by inhibiting glycolysis while promoting fatty acid oxidation, limiting the energy supply required for robust T cell responses^20^. PD1 signaling primarily dampens T cell activation to prevent excessive immune responses that could cause autoimmunity and tissue damage. However, sustained PD1 expression in chronic infections and cancers can lead to T cell exhaustion, characterized by impaired proliferation, cytokine secretion and cytotoxicity^21,22^. Exhausted T cells fail to control chronic pathogens and eliminate tumor cells, allowing immune evasion. Therefore, the recognition that reversing PD1 function restore T cell function from exhausted states has been instrumental in the development of immune checkpoint blockade therapies.

Unlike its well-characterized roles in T cell activation and exhaustion, the function of PD1 in non-T cell populations remains less understood. PD1 is expressed on both human and mouse dendritic cells (DCs). The impact of PD1 on DCs’ antigen presentation, cytokine production and survival have been examined^23^. In vitro, PD1-deficient DCs induce enhanced antigen specific CD8^+^ T cell responses, resulting in increased IFNγ and IL2 secretion compared with PD1-sufficient DCs^23^. In vivo, hepatocellular carcinoma (HCC) intertumoral transfer of PD1-deficient DCs promote CD8 T cell infiltration as well as their cytotoxicity through the increased expression of perforin and granzyme B, enhancing antitumor immunity^23^. The roles of PD1 in DCs were also examined in tumor-infiltrating DCs in ovarian cancer^24,25^. It was found that PD1 blocks NFκB activation via SHP2, which reduces the production of cytokines and prevents proper DC maturation in response to Lipopolysaccharide (LPS) and CpG. It also suppresses antigen presentation through a SHP2 independent mechanism, ultimately weakening T cell activation and promoting tumor immune evasion. The roles of PD1 in DC function are further extended to systemic inflammatory and infectious diseases. In the model of multiple organ dysfunction syndrome induced by zymosan injection, DC surface molecules including CD86, PD1 and PDL1, are highly expressed in splenic DCs, which are accompanied with poor T cell proliferation and IL2 production^26^. Anti-PDL1 antibody treatment to intervene the PD1–PDL1 pathway increases DC production of IL12p70, while decreasing IL12p40 and IL10 production by DCs, restoring T cell proliferation and IL2 production^26^. PD1-dependent DC function was also investigated in LPS-induced DC survival in vivo. LPS stimulated wild-type DCs express PD1 and undergo apoptosis, while PD1-deficient DCs are more resistant to apoptosis^27^. Treating mice with anti-PD1 antibody during DC maturation enhances DC survival. T cell responses generated under these conditions display elevated antigen-specific IFNγ production and proliferation^27^. Mechanistically, PD1 signaling in DCs limits CD40-40L signaling, contributing DC survival^27^. PD1 expressed on DCs also interferes with the innate function of DCs to clear bacterial infection^28^. Chen and colleagues reported that PD1 expression is induced in DCs upon stimulation and that PD1-deficient DCs exhibit the ability to protect mice from lethal *Listeria* infection^28^. PD1-dependent superior protection is also observed even without adaptive immunity, where PD1-deficient DCs produce more IL12 and TNF upon *Listeria monocytogenes* infection^28^. Therefore, PD1 appears to play an important role in negatively regulating DC function.

Mast cells are tissue resident immune cells best known for their roles in allergic inflammation and immune surveillance, mediating inflammation through the release of granules containing histamine, proteases and cytokines and influencing both innate and adaptive immunity^29^. PD1 expression on mast cells was first reported by investigating mastocytosis, a condition characterized by dysregulated mast cell proliferation^30^. Kataoka et al.^31^ examined patients with cutaneous mastocytosis and found that in approximately one-third of the cases, mast cells express PD1^31^. From analyzing human mastocytosis line LAD2 cells, they found that recombinant PDL1 suppresses the growth of the LAD2 cells, in part by activating SHP1/SHP2 and by inhibiting AKT phosphorylation. Therefore, PD1 may regulate mast cell cell growth^31^. Bioinformatics analysis of patients with melanoma uncovers that mast cells are associated with resistance to anti-PD1 immunotherapy. Anti-PD1 antibody induces PD1^+^ mast cell activation, triggering the release of histamine and cytokines^32^. Importantly, inhibiting the release of histamine and cytokines from mast cells substantially improves the efficacy of PD1 immunotherapy in a subcutaneous melanoma model^32^. Mast cells also play an immune regulatory role via PD1. PD1^+^ mast cells directly interact with immature DCs to support their differentiation into tolerogenic subsets. Direct interaction with mast cells downregulates MHCII and increases PDL1 expression in DCs, supporting regulatory T cell development^33^. The tolerogenic DCs developed from interacting with mast cells express high levels of indoleamine-2,3-deoxigenase (IDO), a rate-limiting enzyme catabolizing tryptophan and inhibiting T cell proliferation^33^. Treating mast cells with anti-PD1 antibody or DCs with anti-PDL1/PDL2 antibody abrogates the generation of tolerogenic DCs^33^.

Human and murine B cells express both PD1 and PDL1 upon activation and PD1 inhibits B cell receptor (BCR) signaling and B cell activation^34–37^. Coligation of PD1 with BCR phosphorylates tyrosine in PD1, recruiting SHP2. Recruited SHP2 dephosphorylates proximal signaling molecules of the BCR, leading to inactivation of downstream molecules, such as PI3K and ERK, thereby dampening BCR signaling and reducing B cell activation^35^. Certain B cell subsets are capable of downregulating tumor T cell immunity via PD1. In advanced-stage HCC, TLR4-induced BCL6 induces PD1^+^ regulatory (protumorigenic) B cell subsets. Upon engaging PDL1, these cells acquire regulatory properties and suppress antitumor T cell responses through IL10 production^38^. In addition to suppressive function of PD1 in B cell activation, a recent study from Tangye and colleagues reported that PD1 signaling is essential for optimal memory B cell and antibody responses both in human and mouse^39^.

Mechanistically, PD1- (or PDL1)-deficient B cells express diminished key transcription regulator, cMyc and its target genes necessary for Ig class switch and optimal antibody production^39^. Supporting this, spleens from B cell-specific PD1-deficient mice show disrupted physiologic accumulation of memory B cells, along with reduced numbers of total and memory B cells in both primary and secondary lymphoid tissues. These findings suggest that PD1 on B cells is essential for maintaining B cell homeostasis.

Macrophages are another cell type expressing PD1. In sepsis, PD1 expression in macrophages is critical in protection from the lethality, in part by suppressing bacterial burden and inflammatory cytokine production^40^. PD1 is also known to regulate macrophage differentiation process. In the model of spinal cord injury, PD1 expressed on macrophages/microglia is involved in the cellular polarization process, that is, to classically activated proinflammatory M1 phenotype^41^. PD1 signal suppresses M1 differentiation by diminishing STAT1 activation and supports anti-inflammatory M2 phenotype cell differentiation by enhancing STAT6 activation^41^. Circulating monocytes that infiltrate tumors and differentiate into macrophages in response to the tumor microenvironment are called tumor-associated macrophages (TAMs). PD1 is highly expressed in TAMs in response to IFNγ, MYD88/IL1R signaling and TLR2/3 stimulation^42–44^, inhibiting the phagocytic capacity of M1 phenotype TAMS and impairing their ability to clear tumor cells^45^. PD1 signal also drives M2 phenotype TAMs polarization, promoting an immunosuppressive, tumor supportive environment^46^. PD1 expressed on TAM increases in mouse models of cancer and in primary human cancers, negatively correlated with their phagocytic activity against tumor cells^46^. Blocking the PD1–PDL1 interaction in vivo enhances their phagocytic function, thereby reducing tumor cell growth and prolonging the survival in a macrophage-dependent manner^46,47^. More recently, Rathmell and colleagues reported that PD1 also suppresses glycolytic activity of TAMs^48^. Myeloid cell-specific PD1 deficiency is associated with enhanced TAM glycolysis and antigen presentation ability, leading to enhanced CD8^+^ T cell activity^48^.

From analyzing single-cell RNA sequencing data of bone marrow progenitor cells, Yu et al. reported that innate lymphoid cells (ILCs) express PD1, marking ILC progenitors^49^. PD1 is also an important negative regulator of KLRG1^+^ ILC2 cell function in both humans and mice, as PD1 deficiency increases ILC2 numbers and reduces worm burden during helminth infection^50^. In tumor microenvironments, IL33/ST2 signaling induces PD1 expression in ILC2s, limiting their ability to support DC recruitment by suppressing CCL5 production^51^. PD1 is also constitutively expressed on a subset of ILC3, a key ILC subset important in gut homeostasis maintenance^52^. Lack of PD1 in ILC3s results in reduced IL22 production, which is essential for intestinal barrier function^53^. Lastly, PD1 expression is found in ILC1 type cells, controlling their proliferation and function within tumor environment^54,55^. Interestingly, tumor-derived lactate increases PD1 expression on the ILCs, altering their metabolic programing^56^. In support, PD1-deficient ILC1 display enhanced IFNγ and granzyme B expression, delaying tumor growth^56^.

The recognition that PD1 regulates immune function beyond T cells has profound implications for immunotherapy and immune modulation. Although traditionally studied in the context of T cell exhaustion, PD1 expression on DCs, macrophages, B cells, ILCs and mast cells indicates a broader function in immune suppression. This expanded understanding calls for a reassessment of PD1 blockade therapies, as targeting PD1 may not only reinvigorate T cell function but also modulate the functions of other immune cell populations. Further research is required to clarify the context-dependent roles of PD1 across diverse immune cell types, evaluate the impact of its blockade on non-T cell-mediated immunity and refine therapeutic strategies to enhance antitumor efficacy while minimizing immune-related adverse effects.

## CTLA4

Cytotoxic T-lymphocyte antigen 4 (CTLA4) was first discovered from searching for a cytotoxic surface molecule from cDNA of activated CD8^+^ T cells^57^. Initial screen of CTLA4 mRNA found that its expression is predominantly found in CD28^+^ T cells^58^. Surface CTLA4 expression is not found in resting T cells, and intracellular CTLA4 is mainly localized in endocytic compartments that contain other secretory granules such as perforin^59^. Activation rapidly increases surface CTLA4 expression, which localizes toward the sites of TCR engagements^59^. The avidity that CTLA4 binds its ligand, B7 molecules on antigen presenting cells, is 20-fold greater than that of CD28^60^. It was thus initially thought that CTLA4 might be the second CD28-like costimulatory molecule, based on the observation that anti-CTLA4 antibody increases CD28-induced T cell proliferation^61,62^. However, it was found that crosslinking CTLA4 greatly diminishes T cell proliferation and IL2 production, proposing inhibitory property of CTLA4-derived signals^63^. Mouse model deficient in CTLA4 develop massive lymphoproliferative and fatal multiorgan inflammation, further supporting the importance of CTLA4-derived coinhibitory signal in T cell tolerance and activation in vivo^64^.

CTLA4 associates with tyrosine phosphatase SYP or SHP2 that dephosphorylates TCR signaling molecules, such as the TCRζ chain^65,66^. It was additionally reported that CTLA4 recruits the PIX-GIT2-PAK2 complex via the kinase PKCη, especially in Foxp3^+^ T_reg_ cells, regulating receptor endocytosis, actin dynamics and T cell-APC interaction^67^. CTLA4 mutation found in patients with common variable immunodeficiency leads to decreased CTLA4 protein expression in T_reg_ cells^68^. However, T_reg_ cell numbers remain unaltered in these patients; instead, their suppressive function and their ability to control antigen presenting cell function seem impaired, suggesting that CTLA4 expressed on T^reg^ cells may boost their regulatory functions.

The role of CTLA4 in T_reg_ cells appears to be context dependent as evidenced by several conflicting results. CTLA4 expressed on T_reg_ cells depletes CD80/CD86 from antigen presenting cells by trogocytosis, enabling more PDL1 available to inhibit T cell activity^69^. On the other hand, T_reg_ cells deficient in CTLA4 fail to control diabetes in an adoptive transfer model^70^, although CTLA4-deficient T_reg_ cells are fully capable of preventing colitis or autoimmune inflammation^71,72^. Interestingly, Paterson et al. reported using a T_reg_ cell-specific CTLA4-deficient mouse model that CTLA4 deletion during adulthood does not induce systemic autoimmune inflammation and that these mice instead develop resistance from autoimmune inflammation^73^. This unexpected resistance is attributed to expansion of functionally competent CTLA4-deficient T_reg_ cells and to the compensatory upregulation of other immune inhibitory molecules in conventional T cells^73^, concluding that CTLA4 controls T_reg_ cell expansion and function to regulate conventional T cell activity. The precise mechanisms underlying the opposing functions of CTLA4 in T_reg_ cell activity remain unclear and warrant future investigation.

Myeloid cells, including monocytes and DCs also express CTLA4. Earlier studies demonstrated that CD14^+^ monocytes from human PBMCs constitutively express CTLA4, and similar CTLA4 expression is found in activated myelomonocytic cell line U937^74^. Treating these cells with anti-CTLA4 antibody inhibits the proliferation and upregulation of surface markers, including CD86 and HLA-DR, induced by IFNγ treatment, suggesting that CTLA4 may regulate monocyte-associated immune responses^74^. CTLA4 is also expressed on human monocyte-derived DCs^75,76^. The expression level changes over the maturation process, as the expression is high on freshly isolated monocytes and is downregulated upon differentiation into immature DCs. DCs matured following stimulation with Toll-like receptor (TLR) ligands regain high CTLA4 expression^75^. Agonistic anti-CTLA4 antibody treatment drastically increases IL10 secretion while diminishing inflammatory IL12 secretion, suggesting that CTLA4 expressed on DCs regulates DC functions^75^. CTLA4 on DCs also plays an inhibitory role, as crosslinking of CTLA4 inhibits DC maturation and antigen presentation ability^76^. Moreover, DCs treated with siRNA targeting CTLA4 display enhanced stimulatory activity in vivo^77^. T cell activation and cytokine production is substantially elevated when DCs do not express CTLA4^78^. Halpert et al.^79^ reported an additional mechanism by which DCs exploit immune regulatory functions via CTLA4. DCs constitutively secrete microvesicles containing intracellular CTLA4, which competitively bind B7 molecules on bystander DCs to downregulate surface B7 expression, limiting their ability to activate T cells^79^. Thus, CTLA4 expression on myeloid cells could support tumor immune evasion. Targeting this pathway may improve the efficacy of immune checkpoint blockade therapies. Future studies should focus on elucidating the precise molecular mechanisms of CTLA4 in different myeloid subpopulations and its potential as a therapeutic target in cancer and autoimmune diseases.

Lastly, B cells activated in the presence of IL4 also express CTLA4^80^. CTLA4 engagement in B cells inhibits IgE and IgG1 production. Mechanistically, CTLA4 signaling inhibits germline Cε and Cγ1 gene mRNA expression and transcription factor activation, required for isotype switching^80^. CTLA4 can also be expressed by CD5^+^ B-1a B cells, an innate B cell subset that produces autoreactive natural antibodies^81^. In the absence of CTLA4, B-1a cells exhibit dysregulated immune functions, with transcriptional programs enriched in antigen processing and presentation^81^. Reconstituting B cell-depleted newborn mice with CTLA4-deficient B-1a cells generates B cell chimeras in which conventional B cells are different from reconstituted self-replenishing B-1a cells. CTLA4-deficient B-1a cell reconstitution induces germinal center B cell and follicular helper T cell responses expressing a highly selected repertoire, which are not observed in CTLA4^+^ B-1a cell recipients^81^.

## Lag3

A novel Ig superfamily surface molecule expressed in activated T cells and natural killer (NK) cells, termed Lag3, was discovered in the year 1990^82^. Structurally, it resembles CD4 surface antigen and shares its binding ligand, MHCII^83^. The Lag3 gene is embedded within the CD4 locus, probably generated by gene duplication of the CD4 gene and probably controlled by similar CD4 regulatory elements^84^. It was initially proposed that Lag3 is associated with Th1 type T cell responses, as surface Lag3 expression correlates with IFNγ-producing T cell subsets^85^. Lag3 expression on activated T cells is further upregulated by certain cytokines including IL12 but not by IL4 or IL10^84^, supporting its association with type 1 Immunity. Lag3 indeed contributes to the development of Th1 type T cell responses, by crosslinking MHCII on antigen presenting cells, which stimulates APCs to secrete IL12^86^.

It was then discovered that Lag3 downregulates phorbol ester-activated T cell proliferation and cytokine production^87^. Lag3 is now widely recognized as a crucial inhibitory coreceptor expressed on activated T cells^88^. The observation that Lag3 modulates the activity of CD8 and NK cells, which do not interact with MHCII, suggests the presence of additional ligands. LSECtin, a member of the DC-SIGN family expressed on endothelial cells and melanoma cells, interacts with Lag3, inhibiting tumor specific T cell responses^89^. Galectin-3 (Gal-3), a lectin with the ability to negatively control T cell responses, binds Lag3, and Lag3 expression is required for Gal-3 to suppress CD8 T cell responses^90^. In support, mice deficient in Gal-3 also display enhanced CD8^+^ T cell functions. Fibrinogen-like protein 1 (FGL1) is a liver secreted protein identified as another ligand of Lag3, inhibiting antigen specific T cell responses through binding the Lag3 and blocking the interaction improves antitumor immunity^91^. However, this notion has recently been challenged, as FGL1 binding may be dispensable for Lag3 to inhibit T cell activation and autoimmune disease development^92^. Due to the potent action of Lag3 to modulate CD8^+^ T cell responses, targeting Lag3 is being considered the promising strategy to reverse T cell exhaustion and to restore CD8 immunity. The phase 3 clinical trial treating patients with metastatic melanoma has yielded prolonged patient survival when combined with anti-PD1 therapy^93^. A phase 1 clinical trial utilizing a bispecific antibody targeting both PD1 and Lag3, tebotelimab, is also being conducted^94^.

The immune regulatory roles of Lag3 in T_reg_ cells have garnered significant attention. Germline Lag3 deficiency does not exhibit any defects in T_reg_ cell development nor develop systemic autoimmunity^95^. The first hint of Lag3 in T_reg_ cell function came from an elegant study from Huang et al. that shows Lag3 expression in thymus-derived T_reg_ cells is necessary for their regulatory function and that ectopic Lag3 expression confers the ability to reduce effector T cell proliferation in vitro^96^. It is also shown that Lag3^+^ T_reg_ cells preferentially expand in the PBMCs of patients with cancer and express immunosuppressive cytokines and potent suppressive activity^97^. Lag3-dependent T_reg_ cell function could operate through Lag3-dependent inhibition of DC activation via MHCII^98^. We also reported that Lag3-deficient T_reg_ cells fail to suppress homeostatic proliferation and T cell-induced colitis development and that Lag3 expression in T_reg_ cells is indispensable for in vivo suppressive function in autoimmune inflammation^99^. Importantly, Lag3 programs T_reg_ cell metabolic profiles to support oxidative phosphorylation suitable for optimal suppression^100^. However, some conflicting findings were also reported by Vignali and colleagues, where they demonstrated that Lag3 dampens T_reg_ cell suppressive function especially within the target tissues (islet in this study)^101^. Although the precise mechanism underlying this discrepancy is not clear, it is possible that Lag3 targeting strategy or genetic background may account for the discrepant results. For example, our study targeted the first three exons of the Lag3 gene, the same exons targeted in germline Lag3^−/−^ mouse model generated by Mathis and colleagues^95^, whereas Vignali’s group targeted the exon 7 from which surface Lag3 expression is abolished, while continuing to release soluble Lag3^101^. Also worth noting is their genetic background. Our Lag3-targeted animal model is in C57BL/6 background, while Lag3-targeted model from Vignali and colleagues is in NOD background.

Accumulating evidence suggests that Lag3 can be expressed in various non-T cell populations. The role of Lag3 in DCs has been explored. Lag3 controls the immune stimulatory property of BMDCs^102^. Bone marrow-derived DCs deficient in Lag3 express four times as much TNF-α than Lag3-sufficient BMDCs at basal level, although there is no difference in cytokine expression upon LPS stimulation^102^. Lag3-deficient BMDCS are also more glycolytic utilizing fewer fatty acids for mitochondrial respiration^102^. When it comes to their CD4^+^ T cell priming abilities, Lag3-deficient BMDCs are more potent inducers of Th1 effector differentiation in ex vivo coculture experiments^102^. Plasmacytoid DCs (pDCs) are a unique DC subset implicated as the main source of type 1 interferons^103^. pDCs express a substantial amount of Lag3 mRNA, 10–15 times more than any other T cell population regardless of activation status, suggesting a constitutive expression of Lag3 in pDCs^104^. Interestingly, the role of Lag3 in pDCs appears to be selective as Lag3-deficient pDCs expand more with CpG stimulation compared with control pDCs, while expressing comparable levels of MHCII, TLR9, IDO, CCR5 and CCR7^104^.

Activated B cells also express surface Lag3^105^. B cell Lag3 expression is dependent on T cell-derived soluble factor, suggesting Lag3 as a marker for T cell-induced B cell activation^105^. Lag3 is found on a subset of natural plasma cells that also express other inhibitory receptors such as PDL1 and PDL2. Following *Salmonella* infection, Lag3^+^CD138^+^ cells are found in the spleen, which also highly express IL10^106^. The Lag3^+^ plasma cell subsets display a distinct transcriptome that maintains quiescent state and IL10 expression. Functionally, Lag3^+^ plasma cells influence host resistance against *Salmonella* infection through IL10 production. Upon infection, mice with higher levels of Lag3^+^IL10^+^CD138^+^ plasma cells exhibit a weaker immune response, whereas mice with fewer LAG3^+^ plasma cells show increased levels of memory T cells, suggesting an immunosuppressive role of Lag3 in plasma cells^106^.

Emerging evidence suggests that microglia, the resident macrophages of the central nervous system, also express Lag3^107^. Excessive microglial activation is linked to several neurological disorders, and Lag3 may play a key role in controlling microglial activation. Microglial Lag3 expression is further enhanced by IFNγ via STAT1 signaling^108^. Importantly, Lag3 knockdown in microglia enhances the expression of inducible nitric oxide synthetase by IFNγ, indicating that Lag3 may modulate inflammatory functions of microglia^108^. Microglial Lag3 expression increases in chronic unpredictable stress-exposed mice, a model for depression, whereas electroconvulsive stimulation, a known antidepressant treatment, reduces microglial Lag3 levels^109^. Blocking Lag3 in microglia induces antidepressant effects and enhances neurogenesis, suggesting that Lag3 may be a potential target for antidepressant therapeutics^109^. A separate recent study examining hippocampal sections from post-mortem brains of patients with bipolar disorder who committed suicide uncovers a significant increase of microglial density^110^. Moreover, the proportion of microglia expressing Lag3 is diminished in patients with bipolar disorder that are suicidal, highlighting a potential role of Lag3 in neuroimmune dysfunction^110^.

## Tim3

Searching for a marker distinguishing Th1 and Th2 type effector cells, Kuchroo and colleagues first discovered the Tim3 (encoded by the *Havcr2* gene) as a major surface molecule identifying IFNγ-producing CD4^+^ T cells^111^. Tim3 is an inhibitory coreceptor preventing autoimmunity and promoting immunological tolerance^112^. Functional blockade with antibody or gene deficiency results in exacerbated autoimmune inflammation, whereas Tim3 overexpression ameliorates the disease. Multiple ligands for Tim3 have been identified. Galectin-9, the first identified ligand, binds glycosylated sites on Tim3 and induces apoptosis or dysfunction of T cells^113^. Carcinoembryonic antigen cell adhesion molecule 1 (CEACAM1) is both a *cis* and *trans* ligand important for the tolerance-inducing property of Tim3^114^. T cells from patients with multiple sclerosis and psoriasis express low expression of Tim3 and are resistant to tolerance induction, suggesting that low Tim3 expression supports greater expansion of inflammatory T cells^115,116^.

Tim3 is widely expressed across various immune cell types, including DCs, macrophages, NK cells and mast cells. This broad expression suggests that Tim3 functions not only a regulator of T cell responses but also as a multifaceted immune checkpoint involved in both innate and adaptive immunity. Tim3 expression is often dynamically controlled by environmental cues, that is, cytokines or pathogen-derived signals, and influence processes such as cytokine production, phagocytosis, antigen presentation and immune tolerance.

Recent studies emphasized the importance of Tim3 in the immune regulatory functions of DCs. Tim3 expressed on DCs binds the phosphatidylserine (PtdSer) on apoptotic cells not only to trigger phagocytosis of apoptotic cells but also to cross-present antigens^117–119^. In addition, Tim3 expression is particularly high on type 1 conventional DCs (cDCs1), where Tim3 may exert the inhibitory effects in DCs. The high mobility group box 1 (HMGB1), an alarmin that binds nucleic acids from dying cells and facilitates their recognition by endosomal TLRs, is identified as another Tim3 ligand, and the interaction suppresses downstream signaling pathways that would otherwise promote the production of type I interferons and proinflammatory cytokines^120,121^. This sequestration of HMGB1 by Tim3 effectively reduces DC activation and limits their ability to activate immune responses. In tumor models, Tim3 on BATF3⁺CD103⁺ cDC1s suppresses the production of CXCL9, a chemokine critical for the recruitment of effector CD8⁺ T cells to the tumor microenvironment, and blockade of Tim3 enhances CXCL9 production and improves antitumor immunity^122^. Tim3 expressed on DCs also modulate antitumor immunity through inflammasome activation^123^. Loss of Tim3 in DCs increases reactive oxygen species accumulation and NLRP3 inflammasome activation, supporting stem-like CD8^+^ T cell expansion. Neutralization of IL1 and IL18 abrogates Tim3-deficient DC-derived antitumor immunity^123^. Tim3-dependent DC function is reported by the Bat3, an adapter protein that binds to the Tim3 cytoplasmic domain and interferes with its inhibitory function^124^. By examining Bat3-deficient DC function in autoimmune inflammation model, the authors show that Bat3 deficiency decreases encephalitogenic differentiation while enhances T_reg_ cell development^124^. Thus, Tim3 serves as a checkpoint molecule in DCs, restraining their ability to sense danger signals and limiting their contribution to antitumor immunity. Tim3-dependent DC function is reported by the Bat3, an adapter protein that binds to the Tim3 cytoplasmic domain and interferes with its inhibitory function^124^. By examining Bat3-deficient DC function in autoimmune inflammation model, the authors show that Bat3 deficiency decreases encephalitogenic differentiation while enhances T_reg_ cell development^124^. Thus, Tim3 serves as a checkpoint molecule in DCs, restraining their ability to sense danger signals and limiting their contribution to antitumor immunity.

Analogous to DCs, Tim3 plays important regulatory roles in macrophages. Tim3 is constitutively expressed on CD14^+^ PBMC monocytes. Tim3 knockdown increases TLR-induced IL12 production, demonstrating a regulatory role of Tim3 in innate immunity of myeloid cells^125,126^. Similarly, Tim3 overexpression in macrophages greatly suppress TLR-mediated inflammatory cytokine production^127^. Tim3 also plays a role in modulating TAM function in tumor progression. In the model of HCC, Tim3 expression in TAM is greatly increased, and the expression correlates with higher tumor grades and poor survival of patients with HCC^128^. Tim3 knockdown by siRNA inhibits TAM activation and suppresses HCC cell growth, suggesting a negative role of Tim3 in TAMs. Notably, Tim3–Gal9 interaction could activate *Mycobacterium* *tuberculosis*-infected macrophages for better bactericidal activity via IL1β production^129,130^.

In the brain, microglia express Tim3 and the expression is further increased by LPS stimulation^131^. Tim3 activation increases microglial production of TGFβ and IL1β, while Tim3 blockade decreases microglial phagocytosis of apoptotic neurons, suggesting that Tim3 controls microglial activity^131^. Microglial Tim3 expression is increased following intracerebral hemorrhage, and Tim3 knockdown mitigates intracerebral hemorrhage-induced brain damage accompanied with diminished IL1β secretion. Mechanistically, Tim3 regulates microglial polarization process, namely Tim3 knockdown supports microglial cell differentiation into anti-inflammatory M2-like subsets^132^. Tim3 can also be upregulated in microglia under cerebral hypoxia-ischemia in a HIF1α-dependent manner^133^. Blocking Tim3 by antibody treatment markedly reduces infarct size, neuronal cell death and neutrophil infiltration, and HIF1α deficiency substantially reduces infarct size and neutrophil infiltration. However, Tim3 overexpression is sufficient to reverse neuronal damage in HIF1α-deficient condition, suggesting a key role of Tim3 in regulating hypoxia-induced brain injury.

## TIGIT

TIGIT discovered as immune receptors containing tyrosine-based inhibition motifs is expressed primarily by activated T cells^134,135^. TIGIT binds poliovirus receptor (PVR, also known as CD155) expressed on DCs^136,137^ and the interaction induces IL10 and diminishes IL12p40 production by DCs^138^. T cells from TIGIT-deficient mice display hyperproliferative responses and increased susceptibility to autoimmunity, suggesting a T cell intrinsic inhibitory function^139^. Moreover, a TIGIT-Fc fusion protein inhibits T cell activation and delayed type hypersensitivity reactions by IL10-dependent mechanism. TIGIT is highly expressed on tumor-infiltrating lymphocytes, and blocking TIGIT together with PD1 markedly enhances CD8 T cell activity^140^. In a recent randomized combination therapy targeting both TIGIT (tiragolumab) and PDL1 (atezolizumab) in patients with metastatic non-small cell lung cancer results in great clinical benefit compared to atezolizumab alone^141^. TIGIT is also expressed on NK cells^141^. NK cell subsets that emerge during cytomegalovirus infection, referred to as ‘adaptive NK cells’, are characterized by the expression of activating markers and inflammatory cytokines, along with low levels of TIGIT. Their expansion following cytomegalovirus reactivation is strongly associated with a reduced leukemia relapse^142^. Myeloid-derived suppressor cells inhibit ZAP70/ERK pathways and NK cell cytotoxic activity via a TIGIT-dependent mechanism, and adaptive NK cells expressing lower TIGIT are resistant to myeloid-derived suppressor cell-derived suppression^143^.

TIGIT also directly influences macrophage function. In the bone marrow of patient with acute myeloid leukemia, M2-type macrophage level is increased^144^. These macrophages exhibit elevated expression of immunosuppressive receptors, including TIGIT, although stimulatory receptor CD226 expression is decreased, suggesting impaired immune activation. TIGIT blockade in M2 macrophages led to macrophage repolarization toward proinflammatory M1 phenotype, an increase in stimulatory receptor CD226 expression, and enhanced CD47-mediated phagocytosis, demonstrating that TIGIT on leukemia-associated macrophages can be targeted for therapeutic purposes^144^.

TIGIT is also expressed in B cells, especially Tim1^+^ B cell subsets^145^. B cells from TIGIT-deficient mice produce less IL10. B cell-specific TIGIT-deficient mice develop severe autoimmune inflammation, suggesting a key role of TIGIT expression for B cell-mediated immune tolerance in the central nervous system^145^. TIGIT expressed on B cells interacts with CD155^+^ follicular helper T (T_FH_) cells to suppress T_FH_ cell proliferation and reduced TIGIT expression on B cells causes CCR6^+^ T_FH_ cell expansion in patients with multiple sclerosis, demonstrating a negative feedback loop between TIGIT^+^ B cells and T_FH_ cell in multiple sclerosis pathogenesis^146^. TIGIT is also expressed on human memory B cell subsets that are crucial for immune regulation^147^. B cell-derived TIGIT directly controls T cell and DC functions, suppressing T cell responses. A decrease of TIGIT^+^ memory B cells is associated with elevated donor specific antibody and T_FH_ responses in allograft patients^147^. TIGIT-dependent regulatory functions of B cells are also observed in memory B cell subsets both TIGIT and Tim1^148^. Depending on the expression of TIGIT and/or Tim1, B cells display differential capacity to control T cell responses. For example, TIGIT/Tim1-double negative memory B cells strongly induce T cell proliferation and cytokine production, whereas TIGIT/Tim1-double positive memory T cells display strong regulatory ability to reduce T cell production of inflammatory cytokines. Therefore, the expression of TIGIT along with Tim1 defines a bona fide regulatory B cell subset whose function is dysregulated in patients with multiple sclerosis.

## Concluding remarks

**Fig. 1:**
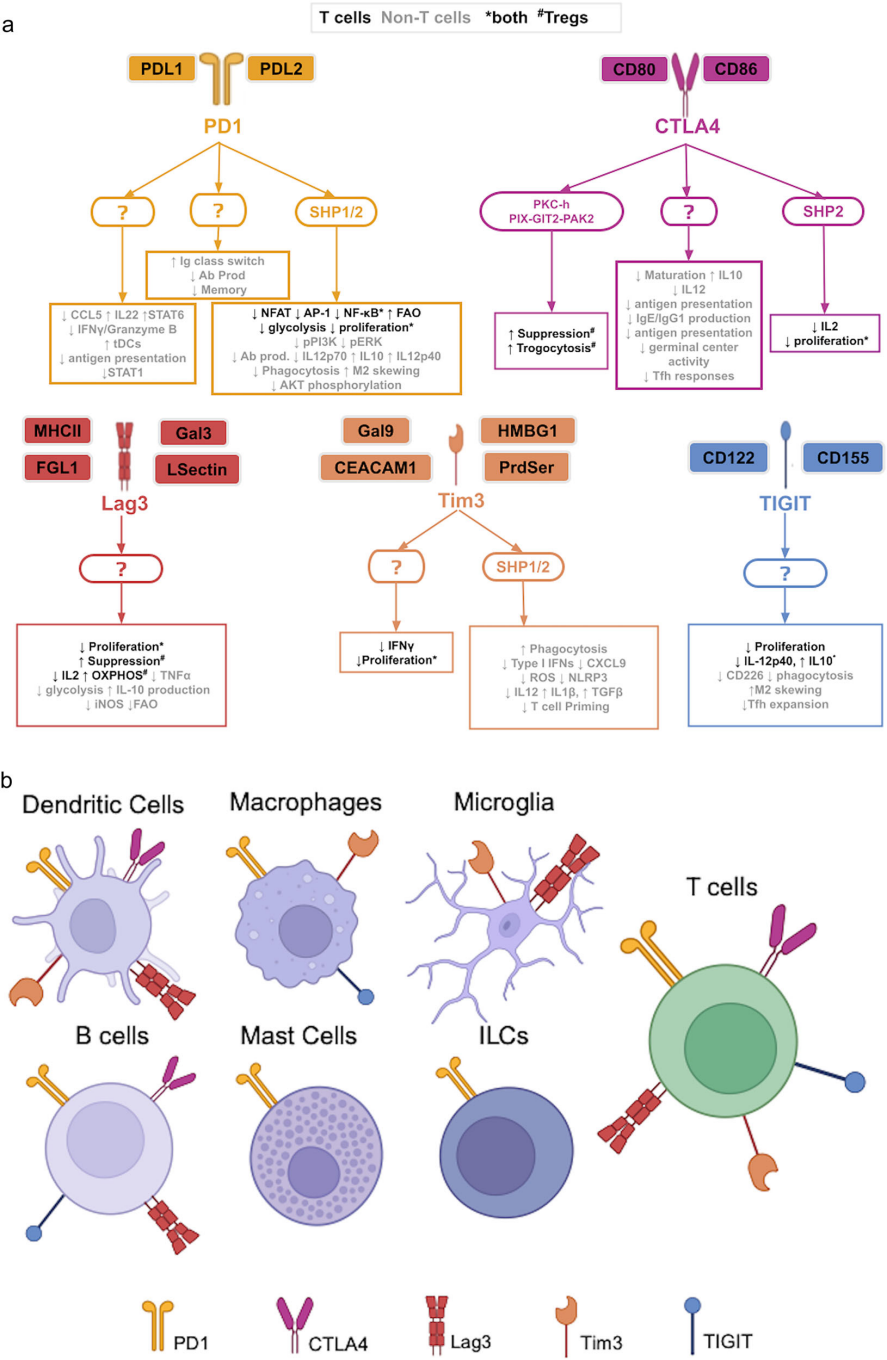
Inhibitory receptor expression in immune cells. **a** This schematic illustrates the ligand–receptor interactions and downstream immunoregulatory effects of five inhibitory receptors: PD1, CTLA4, Lag3, Tim3 and TIGIT. Each receptor interacts with one or more ligands and transduces intracellular signals that lead to diverse function outcomes in a variety of cell types. The question marks indicate that the precises signaling mechanisms or intermediates remain unknown. The black text denotes effects in T cells. The gray text denotes effects in non-T cells (DCs, macrophages, B cells, mast cells and ILCs). The asterisk indicates that the effect is shared between both populations. The pound symbol (#) indicates that the effect is specific to regulatory T cells. Ab prod, antibody production; FAO, fatty acid oxidation; ROS, reactive oxygen species. **b** This diagram illustrates the expression patterns of inhibitory receptors—PD1, CTLA4, Lag3, Tim3 and TIGIT—across immune cell types, T cells, DCs, macrophages, microglia, B cells, mast cells and ILCs.

Although significant advances have been made in the development of immunotherapies targeting coinhibitory receptors on dysfunctional T cells in chronic inflammatory diseases and cancer, enhancing therapeutic efficacy and improving patient response rates remain critical challenges. Increasing evidence indicates that coinhibitory receptors are not restricted to T cells but are also expressed by myeloid lineage cells, including monocytes, macrophages, DCs and microglia, across diverse physiological and pathological contexts (Fig. 1 and Table 1). This broader expression suggests that the current T cell-centric perspective on immune checkpoint modulation may underestimate the wider immunological roles and therapeutic potentials of targeting these receptors.

**Table 1:** Inhibitory coreceptor expression on various non-T cell populations.

Cell type	Expression	Function	Mechanism	References
PD1				
DCs	Upregulated in tumors, inflammation, sepsis	Reduces antigen presentation, cytokine production and survival; inhibits NFκB; promotes apoptosis; and decreases CD8^+^ T cell activation	SHP2-dependent NFκB inhibition, suppresses DC maturation, LPS-induced apoptosis, impairs bacterial clearance and interferes with CD40/MAPK1; ↑ IL10, ↓ IL12p70	^23–28^
Mast cells	Detected in mastocytosis	Regulates proliferation and contributes to immune suppression and resistance to anti-PD1 therapy	SHP1/2 phosphorylation upon PDL1 binding (LAD2 cells), and cell–cell contact induces tolerogenic DCs via IDO	^31–33^
B cells	Upregulated upon activation	Inhibits BCR signaling and activation, supports memory B cell and antibody responses and suppresses antitumor immunity	SHP2 recruitment and dephosphorylation of PI3K and Erk1/2, also supports cMyc-driven class switch, antibody production and memory formation; ↑ IL10	^34–39^
ILCs	Expressed ILC1, ILC2, ILC3 and progenitors	Suppresses ILC function, limits cytokine production and affects gut and tumor immunity	ILC1: PD1 suppresses IFNγ, GzmB, and tumor lactate upregulates PD1 and rewires metabolism ILC2: PD1 suppresses CCL5 and reduces DC recruitment ILC3: PD1 loss ↓ IL22 (gut homeostasis)	^49–56^
Macrophages	Upregulated in sepsis, tumors, spinal cord injury	Suppresses phagocytosis, shifts polarization to M2 (immunosuppressive) and reduces inflammation in sepsis	Inhibits STAT1 (↓M1), promotes STAT6 (↑M2); impairs phagocytosis; suppresses glycolysis and antigen presentation; PD1–PDL1 blockade restores function	^40,48^
CTLA4				
DCs	Expressed in monocyte-derived DCs (upregulated with TLR ligands)	Modulates maturation, cytokine production and antigen presentation	CTLA4 litigation ↓ IL12, ↑ IL10; siRNA knockdown enhances T cell stimulation; vesicle secretion of CTLA4 reduces B7 on neighboring DCs, limiting T cell priming	^75–78^
Monocytes	Constitutively expressed on CD14⁺ cells	Limits activation marker expression and proliferation	Anti-CTLA4 antibody blocks IFNγ-induced upregulation of CD86 and HLA-DR	^74^
B cells	Expressed upon IL4 activation and in B-1a cells	Inhibits class switch recombination and regulates B-1a cell tolerance	Suppresses germline Cε and Cγ1 gene transcription and inhibits transcription factors needed for isotype switching; absence in B-1a → GC and T_FH_ skewing, self-reactivity	^80,81^
Lag3				
DCs	Expressed in BMDCs and highly in pDCs (constitutively, even without stimulation)	Regulates cytokine production and T cell priming	Lag3-deficient DCs → ↑ TNF (baseline); ↓ fatty acid oxidation, ↑ glycolysis; pDCs: Lag3 limits expansion under CpG but not MHCII or TLR9 expression	^102,104^
B cells	Expressed in activated B cells (T cell dependent) and IL10^+^ CD138^+^ plasma cells	Immunosuppressive and limits memory T cell formation after infection	Induced by T cell-derived factors, Lag3^+^ plasma cells express IL10 and maintain quiescence and suppress CD8⁺ T cell memory responses post-infection	^105,106^
Microglia	Expressed and upregulated by IFNγ/STAT1 in mice and expressed in human microglia	Regulates inflammatory activity and may influence neurogenesis and behavior	Knockdown increases inducible nitric oxide synthetase expression, blocking Lag3 has antidepressant-like effects and increases neurogenesis and reduced Lag3^+^ microglia in suicidal bipolar disorder	^107–110^
Tim3				
DCs	Highly expressed on cDC1s and pDCs	Limits inflammation, cross-presentation and cytokine output and inhibits antitumor immunity	Binds PtdSer → phagocytosis and cross-presentation; binds HMGB1 → suppresses TLR responses; limits CXCL9 and inflammasome activity (IL1β, IL18)	^117–121,123^
Macrophages/TAMs	Constitutively expressed in monocytes/macrophages and upregulated in tumors	Suppresses inflammatory cytokine production and promotes tumor progression (for example, HCC)	Tim3 knockdown → ↑ IL12, ↓ TAM activation; Gal9 binding → enhances IL1β in *M. tuberculosis* infection and modulates TLR signaling	^125–130^
Microglia	Basal expression, upregulated by LPS and hypoxia	Modulates neuroinflammation, phagocytosis and polarization and affects brain injury outcomes	↑ TGFβ, IL1β production; knockdown → ↑ M2 polarization, ↓ infarct size/neutrophils; overexpression reverses neuroprotection in HIF1α-deficient models	^131,132^
TIGIT				
Macrophages	Elevated in M2-type macrophages (for example, in acute myeloid leukemia)	Maintains immunosuppressive M2 phenotype and inhibits phagocytosis	TIGIT blockade → repolarization to M1, ↑ CD226, ↑ CD47-mediated phagocytosis	^144^
B cells	Expressed especially in Tim1⁺ and memory B cell subsets	Promotes IL10 production and immune tolerance, regulates T_FH_ cells and suppresses T cell responses	B cell TIGIT interacts with CD155⁺ T_FH_ → inhibits T_FH_ proliferation; TIGIT/Tim1⁺ B cells suppress inflammation; deficiency leads to autoimmunity	^145,147,148^

Current therapeutic antibodies targeting coinhibitory receptors can influence both lymphoid and myeloid compartments, underscoring the importance of elucidating the cell type-specific functions and signaling mechanisms associated with these molecules. A deeper understanding of how coinhibitory receptors regulate immune function in non-T cell populations may reveal novel predictive biomarkers for immunotherapy responsiveness and resistance. Furthermore, dissecting the contributions of myeloid-expressed coinhibitory receptors to immunosuppression, resistance to checkpoint blockade and the emergence of immune-related adverse events could uncover new mechanistic insights and inform the development of more targeted therapeutic strategies.

## References

[1] Chaplin, D. D. Overview of the immune response. *J. Allergy Clin. Immunol.* **125**, S3–S23 (2010). 20176265 10.1016/j.jaci.2009.12.980 PMC2923430

[2] Arneth, B. Molecular mechanisms of immune regulation: a review. *Cells* **14**, 283 (2025). 39996755 10.3390/cells14040283 PMC11853995

[3] Hwang, J.-R. et al. Recent insights of T cell receptor-mediated signaling pathways for T cell activation and development. *Exp. Mol. Med.* **52**, 750–761 (2020). 32439954 10.1038/s12276-020-0435-8 PMC7272404

[4] Chen, L. & Flies, D. B. Molecular mechanisms of T cell co-stimulation and co-inhibition. *Nat. Rev. Immunol.* **13**, 227–242 (2013). 23470321 10.1038/nri3405 PMC3786574

[5] Luckheeram, R. V. et al. CD4⁺ T cells: differentiation and functions. *Clin. Dev. Immunol.* **2012**, 925135 (2012). 22474485 10.1155/2012/925135 PMC3312336

[6] Zhu, J. & Paul, W. E. CD4 T cells: fates, functions, and faults. *Blood* **112**, 1557–1569 (2008). 18725574 10.1182/blood-2008-05-078154 PMC2518872

[7] Trinchieri, G., Pflanz, S. & Kastelein, R. A. The IL-12 family of heterodimeric cytokines: new players in the regulation of T cell responses. *Immunity* **19**, 641–644 (2003). 14614851 10.1016/s1074-7613(03)00296-6

[8] Licona-Limón, P. et al. TH2, allergy and group 2 innate lymphoid cells. *Nat. Immunol.* **14**, 536–542 (2013). 23685824 10.1038/ni.2617

[9] Zhu, J. et al. Stat6 is necessary and sufficient for IL-4’s role in Th2 differentiation and cell expansion. *J. Immunol.* **166**, 7276–7281 (2001). 11390477 10.4049/jimmunol.166.12.7276

[10] Veldhoen, M. et al. TGFβ in the context of an inflammatory cytokine milieu supports de novo differentiation of IL-17-producing T cells. *Immunity* **24**, 179–189 (2006). 16473830 10.1016/j.immuni.2006.01.001

[11] Mangan, P. R. et al. Transforming growth factor-β induces development of the TH17 lineage. *Nature* **441**, 231–234 (2006). 16648837 10.1038/nature04754

[12] Odorizzi, P. M. & Wherry, E. J. Inhibitory receptors on lymphocytes: insights from infections. *J. Immunol.* **188**, 2957–2965 (2012). 22442493 10.4049/jimmunol.1100038 PMC3320038

[13] Blank, C. U. et al. Defining ‘T cell exhaustion’. *Nat. Rev. Immunol.* **19**, 665–674 (2019). 31570879 10.1038/s41577-019-0221-9 PMC7286441

[14] Wherry, E. J. T cell exhaustion. *Nat. Immunol.* **12**, 492–499 (2011). 21739672 10.1038/ni.2035

[15] Ishida, Y. et al. Induced expression of PD-1, a novel member of the immunoglobulin gene superfamily, upon programmed cell death. *EMBO J.* **11**, 3887–3895 (1992). 1396582 10.1002/j.1460-2075.1992.tb05481.x PMC556898

[16] Chamoto, K. et al. Insights from a 30-year journey: function, regulation and therapeutic modulation of PD1. *Nat. Rev. Immunol.* **23**, 682–695 (2023). 37185300 10.1038/s41577-023-00867-9

[17] Boussiotis, V. A. Molecular and biochemical aspects of the PD-1 checkpoint pathway. *N. Engl. J. Med.* **375**, 1767–1778 (2016). 27806234 10.1056/NEJMra1514296 PMC5575761

[18] Sheppard, K.-A. et al. PD-1 inhibits T-cell receptor induced phosphorylation of the ZAP70/CD3ζ signalosome and downstream signaling to PKCθ. *FEBS Lett.* **574**, 37–41 (2004). 15358536 10.1016/j.febslet.2004.07.083

[19] Sharpe, A. H. & Pauken, K. E. The diverse functions of the PD1 inhibitory pathway. *Nat. Rev. Immunol.* **18**, 153–167 (2018). 28990585 10.1038/nri.2017.108

[20] Patsoukis, N. et al. PD-1 alters T-cell metabolic reprogramming by inhibiting glycolysis and promoting lipolysis and fatty acid oxidation. *Nat. Commun.* **6**, 6692 (2015). 25809635 10.1038/ncomms7692 PMC4389235

[21] Jubel, J. M. et al. The role of PD-1 in acute and chronic infection. *Front Immunol.* **11**, 487 (2020). 32265932 10.3389/fimmu.2020.00487 PMC7105608

[22] Jiang, Y., Li, Y. & Zhu, B. T-cell exhaustion in the tumor microenvironment. *Cell Death Dis.* **6**, e1792–e1792 (2015). 26086965 10.1038/cddis.2015.162 PMC4669840

[23] Lim, T. S. et al. PD-1 expression on dendritic cells suppresses CD8^+^ T cell function and antitumor immunity. *Oncoimmunology* **5**, e1085146 (2016). 27141339 10.1080/2162402X.2015.1085146 PMC4839350

[24] Karyampudi, L. et al. PD-1 blunts the function of ovarian tumor-infiltrating dendritic cells by inactivating NF-κB. *Cancer Res*. **76**, 239–250 (2016). 26567141 10.1158/0008-5472.CAN-15-0748 PMC4715980

[25] Krempski, J. et al. Tumor-infiltrating programmed death receptor-1+ dendritic cells mediate immune suppression in ovarian cancer. *J. Immunol.* **186**, 6905–6913 (2011). 21551365 10.4049/jimmunol.1100274 PMC3110549

[26] Liu, Q. et al. Changes in the PD-1 and PD-L1 expressions of splenic dendritic cells in multiple-organ dysfunction syndrome mice and their significance. *Genet Mol. Res*. **13**, 7666–7672 (2014). 25299080 10.4238/2014.September.26.4

[27] Park, S. J. et al. Negative role of inducible PD-1 on survival of activated dendritic cells. *J. Leukoc. Biol.* **95**, 621–629 (2014). 24319287 10.1189/jlb.0813443

[28] Yao, S. et al. PD-1 on dendritic cells impedes innate immunity against bacterial infection. *Blood* **113**, 5811–5818 (2009). 19339692 10.1182/blood-2009-02-203141 PMC2700320

[29] Baran, J. et al. Mast cells as a target-A comprehensive review of recent therapeutic approaches. *Cells* **12**, 1187 (2023). 37190096 10.3390/cells12081187 PMC10136699

[30] Metcalfe, D. D. Mast cells and mastocytosis. *Blood* **112**, 946–956 (2008). 18684881 10.1182/blood-2007-11-078097 PMC2515131

[31] Kataoka, T. R. et al. PD-1 regulates the growth of human mastocytosis cells. *Allergol. Int.* **62**, 99–104 (2013). 23267208 10.2332/allergolint.12-OA-0450

[32] Li, J. et al. PD-1^+^ mast cell enhanced by PD-1 blocking therapy associated with resistance to immunotherapy. *Cancer Immunol. Immunother.* **72**, 633–645 (2023). 36018370 10.1007/s00262-022-03282-6 PMC9947072

[33] Rodrigues, C. P. et al. Tolerogenic IDO^+^ dendritic cells are induced by PD-1-expressing mast cells. *Front Immunol.* **7**, 9 (2016). 26834749 10.3389/fimmu.2016.00009 PMC4724729

[34] Haro, M. A. et al. PD-1 suppresses development of humoral responses that protect against Tn-bearing tumors. *Cancer Immunol. Res*. **4**, 1027–1037 (2016). 27856425 10.1158/2326-6066.CIR-16-0184 PMC5373664

[35] Okazaki, T. et al. PD-1 immunoreceptor inhibits B cell receptor-mediated signaling by recruiting src homology 2-domain-containing tyrosine phosphatase 2 to phosphotyrosine. *Proc. Natl Acad. Sci. USA* **98**, 13866–13871 (2001). 11698646 10.1073/pnas.231486598 PMC61133

[36] Thibult, M. L. et al. PD-1 is a novel regulator of human B-cell activation. *Int Immunol.* **25**, 129–137 (2013). 23087177 10.1093/intimm/dxs098

[37] Wang, X. et al. PD-1-expressing B cells suppress CD4^+^ and CD8^+^ T cells via PD-1/PD-L1-dependent pathway. *Mol. Immunol.* **109**, 20–26 (2019). 30851633 10.1016/j.molimm.2019.02.009

[38] Xiao, X. et al. PD-1hi Identifies a novel regulatory B-cell population in human hepatoma that promotes disease progression. *Cancer Discov.* **6**, 546–559 (2016). 26928313 10.1158/2159-8290.CD-15-1408

[39] Ogishi, M. et al. Impaired development of memory B cells and antibody responses in humans and mice deficient in PD-1 signaling. *Immunity* **57**, 2790–2807.e15 (2024). 39603236 10.1016/j.immuni.2024.10.014 PMC11634639

[40] Huang, X. et al. PD-1 expression by macrophages plays a pathologic role in altering microbial clearance and the innate inflammatory response to sepsis. *Proc. Natl Acad. Sci. USA* **106**, 6303–6308 (2009). 19332785 10.1073/pnas.0809422106 PMC2669369

[41] Yao, A. et al. Programmed death 1 deficiency induces the polarization of macrophages/microglia to the M1 phenotype after spinal cord injury in mice. *Neurotherapeutics* **11**, 636–650 (2014). 24853068 10.1007/s13311-013-0254-x PMC4121443

[42] Bally, A. P. et al. NF-κB regulates PD-1 expression in macrophages. *J. Immunol.* **194**, 4545–4554 (2015). 25810391 10.4049/jimmunol.1402550 PMC4402259

[43] Cho, H.-Y. et al. Interferon-sensitive response element (ISRE) is mainly responsible for IFN-α-induced upregulation of programmed death-1 (PD-1) in macrophages. *Biochimica et. Biophysica Acta* **1779**, 811–819 (2008). 18771758 10.1016/j.bbagrm.2008.08.003

[44] Tartey, S. et al. A MyD88/IL1R axis regulates PD-1 expression on tumor-associated macrophages and sustains their immunosuppressive function in melanoma. *Cancer Res.* **81**, 2358–2372 (2021). 33619117 10.1158/0008-5472.CAN-20-3510 PMC11645125

[45] Kono, Y. et al. Increased PD-1-positive macrophages in the tissue of gastric cancer are closely associated with poor prognosis in gastric cancer patients. *BMC Cancer* **20**, 1–9 (2020). 10.1186/s12885-020-6629-6 PMC7057626 32131763

[46] Gordon, S. R. et al. PD-1 expression by tumour-associated macrophages inhibits phagocytosis and tumour immunity. *Nature* **545**, 495–499 (2017). 28514441 10.1038/nature22396 PMC5931375

[47] Rao, G. et al. Anti-PD-1 induces M1 polarization in the glioma microenvironment and exerts therapeutic efficacy in the absence of CD8 cytotoxic T cells. *Clin. Cancer Res.* **26**, 4699–4712 (2020). 32554515 10.1158/1078-0432.CCR-19-4110 PMC7483850

[48] Bader, J. E. et al. Obesity induces PD-1 on macrophages to suppress anti-tumour immunity. *Nature* **630**, 968–975 (2024). 38867043 10.1038/s41586-024-07529-3 PMC11456854

[49] Yu, Y. et al. Single-cell RNA-seq identifies a PD-1hi ILC progenitor and defines its development pathway. *Nature* **539**, 102–106 (2016). 27749818 10.1038/nature20105

[50] Taylor, S. et al. PD-1 regulates KLRG1^+^ group 2 innate lymphoid cells. *J. Exp. Med* **214**, 1663–1678 (2017). 28490441 10.1084/jem.20161653 PMC5461001

[51] Moral, J. A. et al. ILC2s amplify PD-1 blockade by activating tissue-specific cancer immunity. *Nature* **579**, 130–135 (2020). 32076273 10.1038/s41586-020-2015-4 PMC7060130

[52] Zeng, B. et al. ILC3 function as a double-edged sword in inflammatory bowel diseases. *Cell Death Dis.* **10**, 315 (2019). 30962426 10.1038/s41419-019-1540-2 PMC6453898

[53] Jacquelot, N. et al. PD-1 regulates ILC3-driven intestinal immunity and homeostasis. *Mucosal Immunol.* **17**, 371–386 (2024). 38492744 10.1016/j.mucimm.2024.03.002

[54] Gao, Y. et al. Tumor immunoevasion by the conversion of effector NK cells into type 1 innate lymphoid cells. *Nat. Immunol.* **18**, 1004–1015 (2017). 28759001 10.1038/ni.3800

[55] Heinrich, B. et al. The tumour microenvironment shapes innate lymphoid cells in patients with hepatocellular carcinoma. *Gut* **71**, 1161–1175 (2022). 34340996 10.1136/gutjnl-2021-325288 PMC8807808

[56] Lim, J. X. et al. Programmed cell death-1 receptor-mediated regulation of Tbet^+^NK1.1^−^ innate lymphoid cells within the tumor microenvironment. *Proc. Natl Acad. Sci. USA* **120**, e2216587120 (2023). 37098069 10.1073/pnas.2216587120 PMC10161089

[57] Brunet, J. F. et al. A new member of the immunoglobulin superfamily-CTLA-4. *Nature* **328**, 267–270 (1987). 3496540 10.1038/328267a0

[58] Lindsten, T. et al. Characterization of CTLA-4 structure and expression on human T cells. *J. Immunol.* **151**, 3489–3499 (1993). 8397258

[59] Linsley, P. S. et al. Intracellular trafficking of CTLA-4 and focal localization towards sites of TCR engagement. *Immunity* **4**, 535–543 (1996). 8673700 10.1016/s1074-7613(00)80480-x

[60] Linsley, P. S. et al. CTLA-4 is a second receptor for the B cell activation antigen B7. *J. Exp. Med*. **174**, 561–569 (1991). 1714933 10.1084/jem.174.3.561 PMC2118936

[61] Leach, D. R., Krummel, M. F. & Allison, J. P. Enhancement of antitumor immunity by CTLA-4 blockade. *Science* **271**, 1734–1736 (1996). 8596936 10.1126/science.271.5256.1734

[62] Linsley, P. S. et al. Immunosuppression in vivo by a soluble form of the CTLA-4 T cell activation molecule. *Science* **257**, 792–795 (1992). 1496399 10.1126/science.1496399

[63] Krummel, M. F. & Allison, J. P. CD28 and CTLA-4 have opposing effects on the response of T cells to stimulation. *J. Exp. Med*. **182**, 459–465 (1995). 7543139 10.1084/jem.182.2.459 PMC2192127

[64] Tivol, E. A. et al. Loss of CTLA-4 leads to massive lymphoproliferation and fatal multiorgan tissue destruction, revealing a critical negative regulatory role of CTLA-4. *Immunity* **3**, 541–547 (1995). 7584144 10.1016/1074-7613(95)90125-6

[65] Lee, K. M. et al. Molecular basis of T cell inactivation by CTLA-4. *Science* **282**, 2263–2266 (1998). 9856951 10.1126/science.282.5397.2263

[66] Marengere, L. E. et al. Regulation of T cell receptor signaling by tyrosine phosphatase SYP association with CTLA-4. *Science* **272**, 1170–1173 (1996). 8638161 10.1126/science.272.5265.1170

[67] Kong, K. F. et al. Protein kinase C-eta controls CTLA-4-mediated regulatory T cell function. *Nat. Immunol.* **15**, 465–472 (2014). 24705298 10.1038/ni.2866 PMC4040250

[68] Schubert, D. et al. Autosomal dominant immune dysregulation syndrome in humans with CTLA4 mutations. *Nat. Med*. **20**, 1410–1416 (2014). 25329329 10.1038/nm.3746 PMC4668597

[69] Tekguc, M. et al. T_reg_-expressed CTLA-4 depletes CD80/CD86 by trogocytosis, releasing free PD-L1 on antigen-presenting cells. *Proc. Natl Acad. Sci. USA* **118**, e2023739118 (2021). 34301886 10.1073/pnas.2023739118 PMC8325248

[70] Schmidt, E. M. et al. CTLA-4 controls regulatory T cell peripheral homeostasis and is required for suppression of pancreatic islet autoimmunity. *J. Immunol.* **182**, 274–282 (2009). 19109158 10.4049/jimmunol.182.1.274

[71] Verhagen, J. et al. Enhanced selection of FoxP3^+^ T-regulatory cells protects CTLA-4-deficient mice from CNS autoimmune disease. *Proc. Natl Acad. Sci. USA* **106**, 3306–3311 (2009). 19218450 10.1073/pnas.0803186106 PMC2642665

[72] Read, S. et al. Blockade of CTLA-4 on CD4^+^CD25^+^ regulatory T cells abrogates their function in vivo. *J. Immunol.* **177**, 4376–4383 (2006). 16982872 10.4049/jimmunol.177.7.4376 PMC6108417

[73] Paterson, A. M. et al. Deletion of CTLA-4 on regulatory T cells during adulthood leads to resistance to autoimmunity. *J. Exp. Med*. **212**, 1603–1621 (2015). 26371185 10.1084/jem.20141030 PMC4577848

[74] Wang, X. B. et al. Expression of CTLA-4 by human monocytes. *Scand. J. Immunol.* **55**, 53–60 (2002). 11841692 10.1046/j.0300-9475.2001.01019.x

[75] Laurent, S. et al. CTLA-4 is expressed by human monocyte-derived dendritic cells and regulates their functions. *Hum. Immunol.* **71**, 934–941 (2010). 20650297 10.1016/j.humimm.2010.07.007

[76] Wang, X. B. et al. CTLA4 is expressed on mature dendritic cells derived from human monocytes and influences their maturation and antigen presentation. *BMC Immunol.* **12**, 21 (2011). 21414236 10.1186/1471-2172-12-21 PMC3070687

[77] Ghorbaninezhad, F. et al. CTLA-4 silencing in dendritic cells loaded with colorectal cancer cell lysate improves autologous T cell responses in vitro. *Front Immunol.* **13**, 931316 (2022). 35979362 10.3389/fimmu.2022.931316 PMC9376327

[78] Bakhshivand, M. et al. Boosting immunotherapy efficacy: empowering the potency of dendritic cells loaded with breast cancer lysates through CTLA-4 suppression. *Heliyon* **10**, e37699 (2024). 39309891 10.1016/j.heliyon.2024.e37699 PMC11416247

[79] Halpert, M. M. et al. Dendritic cell-secreted cytotoxic T-lymphocyte-associated protein-4 regulates the T-cell response by downmodulating bystander surface B7. *Stem Cells Dev.* **25**, 774–787 (2016). 26979751 10.1089/scd.2016.0009 PMC4870609

[80] Pioli, C. et al. Inhibition of IgG1 and IgE production by stimulation of the B cell CTLA-4 receptor. *J. Immunol.* **165**, 5530–5536 (2000). 11067906 10.4049/jimmunol.165.10.5530

[81] Yang, Y. et al. CTLA-4 expression by B-1a B cells is essential for immune tolerance. *Nat. Commun.* **12**, 525 (2021). 33483505 10.1038/s41467-020-20874-x PMC7822855

[82] Triebel, F. et al. LAG-3, a novel lymphocyte activation gene closely related to CD4. *J. Exp. Med*. **171**, 1393–1405 (1990). 1692078 10.1084/jem.171.5.1393 PMC2187904

[83] Baixeras, E. et al. Characterization of the lymphocyte activation gene 3-encoded protein. A new ligand for human leukocyte antigen class II antigens. *J. Exp. Med* **176**, 327–337 (1992). 1380059 10.1084/jem.176.2.327 PMC2119326

[84] Bruniquel, D. et al. Regulation of expression of the human lymphocyte activation gene-3 (LAG-3) molecule, a ligand for MHC class II. *Immunogenetics* **48**, 116–124 (1998). 9634475 10.1007/s002510050411

[85] Annunziato, F. et al. Expression and release of LAG-3-encoded protein by human CD4^+^ T cells are associated with IFN-gamma production. *FASEB J.* **10**, 769–776 (1996). 8635694 10.1096/fasebj.10.7.8635694

[86] Avice, M.-N. et al. Lymphocyte activation gene-3, a MHC class II ligand expressed on activated T cells, stimulates TNF-alpha and IL-12 production by monocytes and dendritic cells. *J. Immunol.* **162**, 2748–2753 (1999). 10072520

[87] Huard, B. et al. T cell major histocompatibility complex class II molecules down-regulate CD4^+^ T cell clone responses following LAG-3 binding. *Eur. J. Immunol.* **26**, 1180–1186 (1996). 8647185 10.1002/eji.1830260533

[88] Aggarwal, V., Workman, C. J. & Vignali, D. A. A. LAG-3 as the third checkpoint inhibitor. *Nat. Immunol.* **24**, 1415–1422 (2023). 37488429 10.1038/s41590-023-01569-z PMC11144386

[89] Xu, F. et al. LSECtin expressed on melanoma cells promotes tumor progression by inhibiting antitumor T-cell responses. *Cancer Res*. **74**, 3418–3428 (2014). 24769443 10.1158/0008-5472.CAN-13-2690

[90] Kouo, T. et al. Galectin-3 shapes antitumor immune responses by suppressing CD8^+^ T cells via LAG-3 and inhibiting expansion of plasmacytoid dendritic cells. *Cancer Immunol. Res*. **3**, 412–423 (2015). 25691328 10.1158/2326-6066.CIR-14-0150 PMC4390508

[91] Wang, J. et al. Fibrinogen-like protein 1 is a major immune inhibitory ligand of LAG-3. *Cell* **176**, 334–347e12 (2019). 30580966 10.1016/j.cell.2018.11.010 PMC6365968

[92] Maruhashi, T. et al. Binding of LAG-3 to stable peptide-MHC class II limits T cell function and suppresses autoimmunity and anti-cancer immunity. *Immunity* **55**, 912–924e8 (2022). 35413245 10.1016/j.immuni.2022.03.013

[93] Lipson, E. J. et al. Nivolumab plus Relatlimab in advanced melanoma: RELATIVITY-047 4-year update. *Eur J Cancer* **225**, 115547 (2025). 40513285 10.1016/j.ejca.2025.115547

[94] Luke, J. J. et al. The PD-1- and LAG-3-targeting bispecific molecule tebotelimab in solid tumors and hematologic cancers: a phase 1 trial. *Nat. Med*. **29**, 2814–2824 (2023). 37857711 10.1038/s41591-023-02593-0 PMC10667103

[95] Miyazaki, T. et al. Independent modes of natural killing distinguished in mice lacking Lag3. *Science* **272**, 405–408 (1996). 8602528 10.1126/science.272.5260.405

[96] Huang, C. T. et al. Role of LAG-3 in regulatory T cells. *Immunity* **21**, 503–513 (2004). 15485628 10.1016/j.immuni.2004.08.010

[97] Camisaschi, C. et al. Alternative activation of human plasmacytoid DCs in vitro and in melanoma lesions: involvement of LAG-3. *J. Invest. Dermatol.* **134**, 1893–1902 (2014). 24441096 10.1038/jid.2014.29

[98] Liang, B. et al. Regulatory T cells inhibit dendritic cells by lymphocyte activation gene-3 engagement of MHC class II. *J. Immunol.* **180**, 5916–5926 (2008). 18424711 10.4049/jimmunol.180.9.5916

[99] Do, J. S. et al. An IL-27/Lag3 axis enhances Foxp3^+^ regulatory T cell-suppressive function and therapeutic efficacy. *Mucosal Immunol.* **9**, 137–145 (2016). 26013006 10.1038/mi.2015.45 PMC4662649

[100] Kim, D. et al. Inhibitory co-receptor Lag3 supports Foxp3^+^ regulatory T cell function by restraining Myc-dependent metabolic programming. *Immunity* **57**, 2634–2650.e5 (2024). 39236718 10.1016/j.immuni.2024.08.008 PMC12309520

[101] Zhang, Q. et al. LAG3 limits regulatory T cell proliferation and function in autoimmune diabetes. *Sci. Immunol.* **2**, eaah4569 (2017). 28783703 10.1126/sciimmunol.aah4569 PMC5609824

[102] Garcia Cruz, D. et al. Lymphocyte activation gene-3 regulates dendritic cell metabolic programing and T cell priming function. *J. Immunol.* **207**, 2374–2384 (2021). 34588222 10.4049/jimmunol.2001188 PMC8525871

[103] Reizis, B. Plasmacytoid dendritic cells: development, regulation, and function. *Immunity* **50**, 37–50 (2019). 30650380 10.1016/j.immuni.2018.12.027 PMC6342491

[104] Workman, C. J. et al. LAG-3 regulates plasmacytoid dendritic cell homeostasis. *J. Immunol.* **182**, 1885–1891 (2009). 19201841 10.4049/jimmunol.0800185 PMC2675170

[105] Kisielow, M. et al. Expression of lymphocyte activation gene 3 (LAG-3) on B cells is induced by T cells. *Eur. J. Immunol.* **35**, 2081–2088 (2005). 15971272 10.1002/eji.200526090

[106] Lino, A. C. et al. LAG-3 inhibitory receptor expression identifies immunosuppressive natural regulatory plasma cells. *Immunity* **49**, 120–133.e9 (2018). 30005826 10.1016/j.immuni.2018.06.007 PMC6057275

[107] Galatro, T. F. et al. Transcriptomic analysis of purified human cortical microglia reveals age-associated changes. *Nat. Neurosci.* **20**, 1162–1171 (2017). 28671693 10.1038/nn.4597

[108] Morisaki, Y. et al. LAG-3 expression in microglia regulated by IFN-γ/STAT1 pathway and metalloproteases. *Front Cell Neurosci.* **17**, 1308972 (2023). 38026700 10.3389/fncel.2023.1308972 PMC10663313

[109] Rimmerman, N. et al. Microglia and their LAG3 checkpoint underlie the antidepressant and neurogenesis-enhancing effects of electroconvulsive stimulation. *Mol. Psychiatry* **27**, 1120–1135 (2022). 34650207 10.1038/s41380-021-01338-0

[110] Naggan, L. et al. Suicide in bipolar disorder patients is associated with hippocampal microglia activation and reduction of lymphocytes-activation gene 3 (LAG3) microglial checkpoint expression. *Brain Behav. Immun.* **110**, 185–194 (2023). 36863492 10.1016/j.bbi.2023.02.021

[111] Monney, L. et al. Th1-specific cell surface protein Tim-3 regulates macrophage activation and severity of an autoimmune disease. *Nature* **415**, 536–541 (2002). 11823861 10.1038/415536a

[112] Sanchez-Fueyo, A. et al. Tim-3 inhibits T helper type 1-mediated auto- and alloimmune responses and promotes immunological tolerance. *Nat. Immunol.* **4**, 1093–1101 (2003). 14556005 10.1038/ni987

[113] Zhu, C. et al. The Tim-3 ligand galectin-9 negatively regulates T helper type 1 immunity. *Nat. Immunol.* **6**, 1245–1252 (2005). 16286920 10.1038/ni1271

[114] Huang, Y.-H. et al. CEACAM1 regulates TIM-3-mediated tolerance and exhaustion. *Nature* **517**, 386–390 (2015). 25363763 10.1038/nature13848 PMC4297519

[115] Kanai, Y. et al. Impaired expression of Tim-3 on Th17 and Th1 cells in psoriasis. *Acta Derm. Venereol.* **92**, 367–371 (2012). 22294262 10.2340/00015555-1285

[116] Koguchi, K. et al. Dysregulated T cell expression of TIM3 in multiple sclerosis. *J. Exp. Med*. **203**, 1413–1418 (2006). 16754722 10.1084/jem.20060210 PMC2118310

[117] Nakayama, M. et al. Tim-3 mediates phagocytosis of apoptotic cells and cross-presentation. *Blood* **113**, 3821–3830 (2009). 19224762 10.1182/blood-2008-10-185884

[118] DeKruyff, R. H. et al. T cell/transmembrane, Ig, and mucin-3 allelic variants differentially recognize phosphatidylserine and mediate phagocytosis of apoptotic cells. *J. Immunol.* **184**, 1918–1930 (2010). 20083673 10.4049/jimmunol.0903059 PMC3128800

[119] Santiago, C. et al. Structures of T cell immunoglobulin mucin protein 4 show a metal-Ion-dependent ligand binding site where phosphatidylserine binds. *Immunity* **27**, 941–951 (2007). 18083575 10.1016/j.immuni.2007.11.008 PMC2330274

[120] Chiba, S. et al. Tumor-infiltrating DCs suppress nucleic acid–mediated innate immune responses through interactions between the receptor TIM-3 and the alarmin HMGB1. *Nat. Immunol.* **13**, 832–842 (2012). 22842346 10.1038/ni.2376 PMC3622453

[121] de Mingo Pulido, Á et al. The inhibitory receptor TIM-3 limits activation of the cGAS-STING pathway in intra-tumoral dendritic cells by suppressing extracellular DNA uptake. *Immunity* **54**, 1154–1167.e7 (2021). 33979578 10.1016/j.immuni.2021.04.019 PMC8192496

[122] de Mingo Pulido, Á et al. TIM-3 regulates CD103+ dendritic cell function and response to chemotherapy in breast cancer. *Cancer cell* **33**, 60–74. e6 (2018). 29316433 10.1016/j.ccell.2017.11.019 PMC5764109

[123] Dixon, K. O. et al. TIM-3 restrains anti-tumour immunity by regulating inflammasome activation. *Nature* **595**, 101–106 (2021). 34108686 10.1038/s41586-021-03626-9 PMC8627694

[124] Tang, R. et al. Tim-3 adapter protein Bat3 acts as an endogenous regulator of tolerogenic dendritic cell function. *Sci. Immunol.* **7**, eabm0631 (2022). 35275752 10.1126/sciimmunol.abm0631 PMC9273260

[125] Zhang, Y. et al. Tim-3 regulates pro- and anti-inflammatory cytokine expression in human CD14+ monocytes. *J. Leukoc. Biol.* **91**, 189–196 (2012). 21844165 10.1189/jlb.1010591 PMC3290426

[126] Zhang, Y. et al. Tim-3 negatively regulates IL-12 expression by monocytes in HCV infection. *PLoS One* **6**, e19664 (2011). 21637332 10.1371/journal.pone.0019664 PMC3102652

[127] Yang, X. et al. T cell Ig mucin-3 promotes homeostasis of sepsis by negatively regulating the TLR response. *J. Immunol.* **190**, 2068–2079 (2013). 23365080 10.4049/jimmunol.1202661

[128] Yan, W. et al. Tim-3 fosters HCC development by enhancing TGF-beta-mediated alternative activation of macrophages. *Gut* **64**, 1593–1604 (2015). 25608525 10.1136/gutjnl-2014-307671

[129] Sada-Ovalle, I. et al. The Tim3-galectin 9 pathway induces antibacterial activity in human macrophages infected with Mycobacterium tuberculosis. *J. Immunol.* **189**, 5896–5902 (2012). 23180819 10.4049/jimmunol.1200990 PMC3516679

[130] Jayaraman, P. et al. Tim3 binding to galectin-9 stimulates antimicrobial immunity. *J. Exp. Med*. **207**, 2343–2354 (2010). 20937702 10.1084/jem.20100687 PMC2964580

[131] Wang, H. W. et al. Microglia activity modulated by T cell Ig and mucin domain protein 3 (Tim-3). *Cell Immunol.* **293**, 49–58 (2015). 25557503 10.1016/j.cellimm.2014.12.005

[132] Chen, Z. Q. et al. Negative regulation of glial Tim-3 inhibits the secretion of inflammatory factors and modulates microglia to antiinflammatory phenotype after experimental intracerebral hemorrhage in rats. *CNS Neurosci. Ther.* **25**, 674–684 (2019). 30677253 10.1111/cns.13100 PMC6515709

[133] Koh, H. S. et al. The HIF-1/glial TIM-3 axis controls inflammation-associated brain damage under hypoxia. *Nat. Commun.* **6**, 6340 (2015). 25790768 10.1038/ncomms7340 PMC4383004

[134] Yu, X. et al. The surface protein TIGIT suppresses T cell activation by promoting the generation of mature immunoregulatory dendritic cells. *Nat. Immunol.* **10**, 48–57 (2009). 19011627 10.1038/ni.1674

[135] Chauvin, J. M. & Zarour, H. M. TIGIT in cancer immunotherapy. *J. Immunother. Cancer* **8**, e000957 (2020). 32900861 10.1136/jitc-2020-000957 PMC7477968

[136] Chiang, E. Y. & Mellman, I. TIGIT-CD226-PVR axis: advancing immune checkpoint blockade for cancer immunotherapy. *J. Immunother. Cancer* **10**, e004711 (2022). 35379739 10.1136/jitc-2022-004711 PMC8981293

[137] Boles, K. S. et al. A novel molecular interaction for the adhesion of follicular CD4 T cells to follicular DC. *Eur. J. Immunol.* **39**, 695–703 (2009). 19197944 10.1002/eji.200839116 PMC3544471

[138] Yue, C. et al. TIGIT as a promising therapeutic target in autoimmune diseases. *Front Immunol.* **13**, 911919 (2022). 35720417 10.3389/fimmu.2022.911919 PMC9203892

[139] Joller, N. et al. Cutting edge: TIGIT has T cell-intrinsic inhibitory functions. *J. Immunol.* **186**, 1338–1342 (2011). 21199897 10.4049/jimmunol.1003081 PMC3128994

[140] Inozume, T. et al. Melanoma cells control antimelanoma CTL responses via interaction between TIGIT and CD155 in the effector phase. *J. Invest Dermatol* **136**, 255–263 (2016). 26763445 10.1038/JID.2015.404

[141] Wang, F. et al. TIGIT expression levels on human NK cells correlate with functional heterogeneity among healthy individuals. *Eur. J. Immunol.* **45**, 2886–2897 (2015). 26171588 10.1002/eji.201545480

[142] Cichocki, F. et al. CD56dimCD57^+^NKG2C^+^ NK cell expansion is associated with reduced leukemia relapse after reduced intensity HCT. *Leukemia* **30**, 456–463 (2016). 26416461 10.1038/leu.2015.260 PMC4740203

[143] Sarhan, D. et al. Adaptive NK cells with low TIGIT expression are inherently resistant to myeloid-derived suppressor cells. *Cancer Res*. **76**, 5696–5706 (2016). 27503932 10.1158/0008-5472.CAN-16-0839 PMC5050142

[144] Brauneck, F. et al. TIGIT blockade repolarizes AML-associated TIGIT^+^ M2 macrophages to an M1 phenotype and increases CD47-mediated phagocytosis. *J. Immunother. Cancer* **10**, e004794 (2022). 36549780 10.1136/jitc-2022-004794 PMC9791419

[145] Xiao, S. et al. Checkpoint receptor TIGIT expressed on Tim-1^+^ B cells regulates tissue inflammation. *Cell Rep.* **32**, 107892 (2020). 32668241 10.1016/j.celrep.2020.107892 PMC7496220

[146] Asashima, H. et al. Impaired TIGIT expression on B cells drives circulating follicular helper T cell expansion in multiple sclerosis. *J. Clin. Invest* **132**, e156254 (2022). 36250467 10.1172/JCI156254 PMC9566906

[147] Hasan, M. M. et al. Implication of TIGIT^+^ human memory B cells in immune regulation. *Nat. Commun.* **12**, 1534 (2021). 33750787 10.1038/s41467-021-21413-y PMC7943800

[148] Varghese, J. F. et al. Human regulatory memory B cells defined by expression of TIM-1 and TIGIT are dysfunctional in multiple sclerosis. *Front Immunol.* **15**, 1360219 (2024). 38745667 10.3389/fimmu.2024.1360219 PMC11091236

